# Rapidly recurring massive pleural effusion as the initial presentation of sarcoidosis: A case report

**DOI:** 10.1097/MD.0000000000024027

**Published:** 2021-02-12

**Authors:** Mutaz Albakri, Mushtaq Ahmad, Mouhand F.H. Mohamed

**Affiliations:** Internal Medicine Department, Hamad Medical Corporation, Doha, Qatar.

**Keywords:** case report, large pleural effusion, massive pleural effusion, pleurodesis, sarcoid, steroids

## Abstract

**Rationale:**

Sarcoidosis is a multisystem granulomatous disease with unknown etiology. It affects mainly the lungs, but it can affect almost any other organ. Nevertheless, pleural involvement with the development of pleural effusion is relatively rare. It is usually mild and responsive to treatment with systemic steroids. Here we present a case of rapidly recurring massive unilateral pleural effusion caused by sarcoidosis that was resistant to systemic steroids.

**Patient concerns:**

A 55-year-old lady presented with shortness of breath of 2-months duration. No other respiratory symptoms were reported. On physical examination, there were signs of left-sided pleural effusion, splenomegaly, and inguinal lymph nodes. These findings were confirmed by chest x-ray showing massive pleural effusion. Work up of the effusion revealed an exudative effusion with lymphocyte predominance. Pan-computed tomography scan revealed multiple thoracic, abdominal and inguinal lymphadenopathy; additionally, a left-sided pleural effusion and an enlarged spleen; that contained variable hypodense nodular lesions. Positron emission tomography-computed tomography showed intense uptake in the spleen and the lymph nodes. Inguinal lymph node biopsy showed non-necrotizing granulomatous inflammation. Due to suspicion of malignancy, left medical thoracoscopy was done, and biopsy of the parietal pleura showed nonspecific inflammation without evidence of malignancy or tuberculosis.

**Diagnosis:**

Sarcoidosis was diagnosed based on the finding of the non-necrotizing granulomatous inflammation with no evidence of malignancy or infection on several microbiological and pathological samples.

**Interventions:**

The patient was treated with repeated pleural fluid drainage. Steroids failed to prevent pleural effusion recurrence. Surgical left side pleurodesis was eventually performed.

**Outcomes:**

At more than 1 year follow up, the patient showed no recurrence of pleural effusion or development of any other symptoms.

**Lessons:**

Sarcoidosis may rarely present with massive pleural effusion, as this presentation is rare; it is imperative to rule out other causes of massive pleural effusion. Massive pleural effusion in sarcoidosis may be steroid-resistant. Pleurodesis may have a role in such a scenario.

## 1 Introduction

Sarcoidosis is a multisystem granulomatous disease with unknown etiology.^[1]^ It frequently presents with hilar lymphadenopathy, lung infiltrates and skin and ocular lesions. It may also affect many other organs including rarely the pleura.^[2]^ The disease is usually diagnosed based on the constellation of history, physical examination, radiological findings, and histopathological confirmation of granulomatous inflammation along with the exclusion of other causes.^[3]^

Pleural involvement, when occurs, may manifest in the form of pleural thickening, pleural effusion (PE) and pneunmothorax. PE is reported in only around 1% in case series of patients.^[4]^ It is usually mild and responsive to steroids. Massive PE caused by sarcoidosis is extremely rare and has been responsive to treatment with systemic steroids according to 1 report.^[5]^ The literature is scarce about the best treatment option for sarcoidosis-induced PE when resistant to steroids. Here we present a case of massive recurrent unilateral PE caused by sarcoidosis that is resistant to systemic steroids.

## 2 Case report

A 55 years old lady attended the emergency department complaining of shortness of breath of 2-months duration. The shortness of breath was insidious in onset and followed a progressive course. Besides, she reported orthopnea, primarily upon lying on her right side (trepopnea). The patient denied; cough, chest pain, wheezes, or fever. There was no history of joint pain, skin rash, photosensitivity, or other symptoms suggestive of connective tissue disease. The patient was a lifelong nonsmoker. She is a housewife with no relevant occupational exposure.

On examination, she was distressed and tachypneic. Her oxygen saturation measured 91%, and the trachea was deviated to the right. No Jugular venous distension was present. The chest examination was consistent with left-sided PE, and the abdominal exam showed splenomegaly. She had palpable bilateral inguinal lymph nodes. There was no lower limb edema. No rash was noticed over the chins.

**Figure 1:**
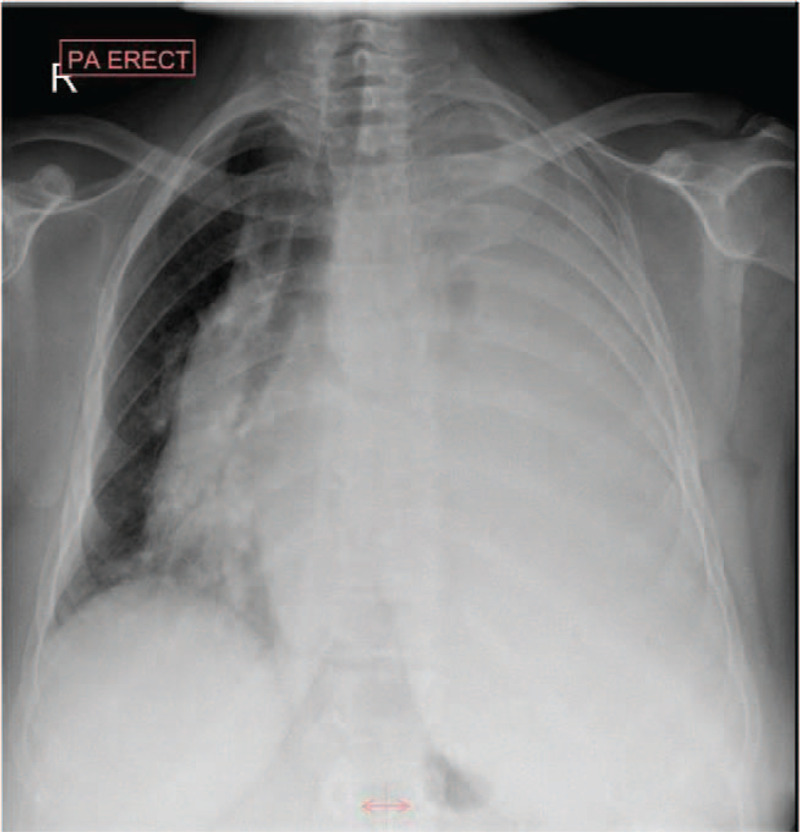
Chest X ray at presentation showed complete opacification of the left hemithorax consistent with massive pleural effusion. The mediastinum appears shifted to the right side.

Initial laboratory evaluation revealed mild normocytic anemia with no other abnormalities on cell counts. Erythrocyte sedimentation rate (ESR) was elevated. Chemistry showed a normal kidney and liver functions. C-reactive protein (CRP) was 22.2 mg/L (0–5 mg/L), and procalcitonin was not elevated. The serum angiotensin converting enzyme level was raised. Chest X-ray revealed complete white-out of the left hemothorax suggestive of massive PE; additionally, a contralateral mediastinal shift (Fig. 1). Therapeutic and diagnostic pleural tapping was performed. The pleural fluid analysis revealed an exudative effusion with lymphocyte predominance (Table 1). Adenosine deaminase was 2.0 U/L. Connective tissue disease (CTD) screening panel, including antinuclear antibody, antinuclear cytoplasmic antibody, rheumatoid factor, and anti citrullinated c peptides were negative.

**Table 1:** Pleural fluid analysis (cell differential and chemistry) results.

Investigation	Result	Reference range
WBC^∗^	225/uL	<1000/uL
RBC	200/uL	0–10,000/uL
LDH	111 U/L	<200 U/L
pH	7.54	7.60–7.66
Protein^†^	61 g/L	<25 g/L
Glucose	16.3 mmol/L	4.0–5.4 mmol/L

LDH = lactate dehydrogenase, RBC = red blood cells, WBC = white blood cells.

∗ Lymphocyte predominance 89%, neutrophils 5%, eosinophils 3%, and mesothelial cells 3%.

† High protein suggestive of exudative pleural effusion.

**Figure 2:**
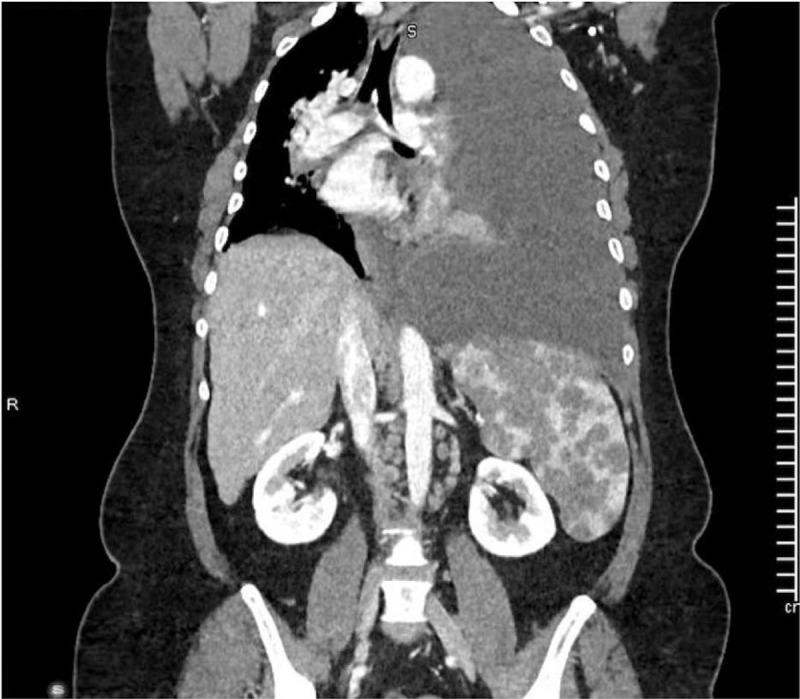
Chest and abdomen CT scan (coronal view) showed massive left sided pleural effusion, mediastinal shifting, multiple splenic hypo-attenuated nodular lesions along with multiple abdominal paraaortic lymph nodes. CT = computed tomography.

**Figure 3:**
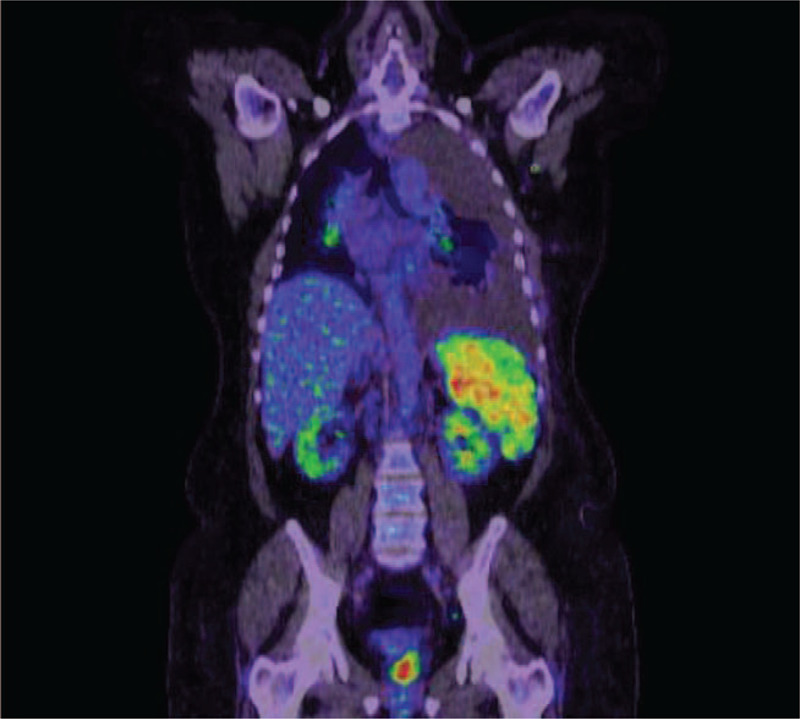
PET-CT scan showed increased pathological FDG uptake involving lymph nodes above and below the diaphragm in addition to the spleen. PET-CT = Positron emission tomography-computed tomography.

Two cytological evaluation and flow cytometry of the pleural fluid showed no evidence of malignant cells. Chest, abdomen, and pelvic computed tomography (CT) scan demonstrated multiple thoracic, abdominal, and inguinal lymph nodes along with left-sided PE. The spleen was enlarged with variable size hypodense lesions (Fig. 2). CT guided core biopsy of the right inguinal lymph node showed the pathological findings of non-necrotizing granulomatous inflammation. Positron emission tomography-CT was requested and showed intense fluorodeoxyglucose (FDG) uptake in the spleen and the lymph nodes with no other areas of uptake (Fig. 3). The initial histopathological evaluation showed evidence of non-necrotizing granulomatous inflammation suggestive of sarcoidosis; however, the presence of massive PE pushed towards the need to rule out other possibilities mainly; tuberculosis (TB) and malignancy. Left side medical thoracoscopy (pleuroscopy) was performed that showed erythematous inflammation of the parietal pleura. Histopathology report showed only non-specific inflammation with no evidence of malignancy. All microbiological tests were negative.

In light of the mentioned findings, we labeled the patient as sarcoidosis. She was started on prednisolone 40 mg daily and was discharged home for regular follow up in the outpatient clinic. Over the next 3 weeks, the patient had a rapid recurrence of the effusion, necessitating the need for therapeutic drainage twice. Because of recurrent rapid re-accumulation and failure of medical treatment, the patient was referred to the thoracic surgeon, and surgical pleurodesis was performed without complications.

Later evaluation with pulmonary function tests revealed a normal lung function and a 6-minute walk test. At more than 1 year follow up, the patient showed no recurrence of PE or development of any symptoms.

## 3 Discussion

The diagnostic process of PE in the absence of heart, liver, or kidney disease usually starts with a diagnostic pleural fluid analysis to differentiate between transudative and exudative PE, which helps narrow down the differential diagnosis. Empyema, TB, and malignancy are common causes of undiagnosed exudative PE.^[6]^ Microbiological studies help in ruling out infectious causes. Cytopathological analysis can often give positive results in 60% of malignant causes.^[7]^ However, if no definite diagnosis is reached, it is recommended to proceed with a pleural biopsy sampling (blind, or thoracoscopy) to rule out sinister causes such as TB and malignancy. Additionally, to look for rarer etiologies.^[8]^ Massive PE is defined as a PE that causes complete or near-complete hemithorax opacification on chest imaging.^[9]^ The common causes of massive PE are limited.^[9]^ Studies from developed countries have revealed that malignancy accounts for the majority of massive PE cases accounting for 53% to 67%, followed by parapneumonic effusion with an estimated prevalence of 8% to 23%, then liver cirrhosis 6% to 9% and TB 4% to 10% among other limited causes.^[10,11]^ Studies from the developing world showed that malignancy (38%) and TB (25%–45%) are the most common causes followed by parapneumonic effusion, with the 3 causes combined causing 83% to 89% of the cases (TB was the most common cause in Africa).^[9–12]^

None of the mentioned earlier mentioned studies examining the causes of massive PE reported pleural sarcoidosis as a cause.^[9–12]^ PE is unusual in sarcoidosis, and when present, it is usually mild to moderate.^[4,13–18]^ When reviewing the literature, we found that the prevalence of sarcoidosis-related PE ranges between 0% and 4% in 7 studies with a total of 974 patients; we calculated the prevalence using the raw count data from these studies, and we estimated it to be 1.6%.^[4,13–18]^ It seems that the sensitivity of diagnosing PE increases with CT scan, as demonstrated by Szwarcberg et al in a study examining 61 patients, and it revealed that the frequency of PE was 8%.^[19]^ However, it is worthy to note that the PE was not caused directly by sarcoidosis in all these cases, so the prevalence of true sarcoid-related effusion may be less than the earlier mentioned estimates. Keeping this uncertainty in mind, when a clinician is faced with a patient with sarcoidosis and a PE, a careful look for an underlying disease is needed, this is especially true when faced with a large or massive effusion.^[4]^

Our patient had a massive exudative PE, not responding to initial steroids. Due to the rapidity of recollection despite multiple trials of therapeutic drainage, our patient needed pleurodesis, which helped to control her effusion. She remained free of symptoms on follow-up, not needing any further medications. Features that were supportive of sarcoidosis-related PE were; the presence of hilar lymph nodes, a raised angiotensin converting enzyme level, and non-necrotizing granuloma findings. Other causes of massive PE in our patient (TB, malignancy and PPE) were deemed less likely given; the absence of constitutional symptoms, negative cytology, histopathology, TB work-up, and very low pleural adenosine deaminase level; additionally, the stability of her symptoms on follow-up for more than a year. Gallium scan with the finding of Lambda and Panda pattern may further support the diagnosis of sarcoidosis.^[20]^ Limited by suboptimal sensitivity and specificity, and the strong likelihood of sarcoidosis in our case, we deemed it unnecessary in our patient. The presence of PE is an unusual feature of sarcoidosis. Furthermore, massive PE is a rare presentation of sarcoidosis that has been reported only a few times in the literature.^[21–26]^ Our case is unique because; the PE was the first and only presenting feature of sarcoidosis; the PE was massive; our patient needed pleurodesis which led to remission of the effusion, a treatment that may be considered in similar instances; we did an extensive work-up to rule out other causes of PE, and we followed the patient for a long time ensuring the absence of other etiologies mimicking sarcoidosis. The patient will continue to follow in our clinic, we will observe for development of other sarcoidosis features that may necessitate treatment.

## 4 Conclusions

Although unusual, sarcoidosis can present with PE; however, when present, it is usually mild to moderate. Our case highlights that sarcoidosis can rarely present as a massive PE, and this can be the presenting or only feature of the disease. Clinicians must be aware of the low prevalence of sarcoidosis related PE, therefore, ruling out other diagnoses before attributing PE to sarcoidosis is imperative. If no other etiologies detected, however, these patients should be followed closely for the development of any new symptoms or signs hinting towards another underlying disease. Our case also sheds light on the role of pleurodesis as a potential therapeutic modality in sarcoid patients with massive PE failing to respond to medical therapy.

### 4.1 Learning points

- The leading causes of massive PE worldwide are malignancy, TB, and parapneumonic effusion.
- Massive PE is a very rare manifestation of sarcoidosis. Thus, other causes of PE (especially malignancy, PPE, and TB) should be ruled out before attributing the PE to sarcoidosis.
- Pleurodesis role on massive PE failing to respond to medical therapy is to be further explored.

## Acknowledgment

The authors want to acknowledge all medical personnel who took part in the care of this patient. Qatar National Library funded the publication of this paper.

## Author contributions

MAB and MFHM conceived the idea of the case report. MAB wrote the case presentation and constructed the table and figures. MFHM wrote the discussion part. MAB, MFHM, and MA reviewed the initial manuscript. Finally, the manuscript was finalized and approved by all authors for submission and publication.

## Abbreviations

Abbreviations: Chest XR = chest x-ray, CT = computed tomography, PE = pleural effusion, PPE = parapneumonic effusion, TB = tuberculosis.
